# Metagenomic and transcriptomic investigation of pediatric acute liver failure cases reveals a common pathway predominated by monocytes

**DOI:** 10.1128/mbio.03913-24

**Published:** 2025-03-18

**Authors:** Ruben H. de Kleine, Ellen C. Carbo, Willem S. Lexmond, Xuewei W. Zhou, Alicia de Kroon, Hailiang Mei, Sander T. H. Bontemps, Rick Hennevelt, Lilli Gard, Igor A. Sidorov, Stefan A. Boers, Marius C. van den Heuvel, Emilie P. Buddingh, Aloys C. M. Kroes, Vincent E. de Meijer, Elisabeth H. Schölvinck, Karin J. von Eije, Simon P. Jochems, Jutte J. C. de Vries

**Affiliations:** 1 Department of Surgery, Section of Hepatobiliary Surgery and Liver Transplantation, University of Groningen, University Medical Center Groningen 3647 , Groningen, The Netherlands; 2 Leiden University Center for Infectious Diseases (LUCID), Leiden University Medical Center 4501 , Leiden, The Netherlands; 3 Department of Pediatrics, Section of Paediatric Gastroenterology and Hepatology, Beatrix Children’s Hospital, University of Groningen, University Medical Center Groningen 571087 , Groningen, The Netherlands; 4 Department of Medical Microbiology, University of Groningen, University Medical Center Groningen 3647 , Groningen, Groningen, The Netherlands; 5 Sequencing Analysis Support Core, Department of Biomedical Data Sciences, Leiden University Medical Center 568306 , Leiden, The Netherlands; 6 Department of Pediatric Intensive Care, Beatrix Children’s Hospital, University of Groningen, University Medical Center Groningen 571087 , Groningen, The Netherlands; 7 GenomeScan B.V., Leiden, The Netherlands; 8 Department of Pathology, University of Groningen, University Medical Center Groningen 3647 , Groningen, The Netherlands; 9 Willem-Alexander Children’s Hospital, Department of Pediatrics, Leiden University Medical Center 568649 , Leiden, The Netherlands; 10 Department of Pediatrics, Section Infectious Diseases and Immunology, University of Groningen, University Medical Center Groningen 3647 , Groningen, The Netherlands; Institute of Microbiology and Immunology, Ljubljana, Slovenia

**Keywords:** metagenomics, transcriptomics, adeno-associated virus, childhood hepatitis

## Abstract

In 2022, a cluster of severe childhood hepatitis was detected primarily in Europe and North America, leading to a global alert by the World Health Organization. An association with adeno-associated virus 2 (AAV2) in conjunction with human adenoviruses was found. Five percent of the cases progressed to acute liver failure, necessitating transplantation. The mechanism of disease that accounts for fulminant liver failure in these patients remains incompletely described. An upsurge was observed of in the five total cases of acute liver failure that presented to the Dutch national referral center for pediatric liver transplantation in the spring of 2022. An in-depth molecular analysis of the mechanism of pediatric acute liver failure was performed using targeted transcriptomics and metagenomics to identify any virus present in the cases, immune profile haplotypes, and differentially expressed gene groups. Explanted liver tissue and plasma samples (*n* = 15) were subjected to viral metagenomic and human transcriptomic profiling, targeting >600 inflammatory genes. Liver transcriptomic signatures of transplanted cases were compared with those of pediatric controls from a liver biobank (*n* = 6). AAV2, adenoviruses, and herpesviruses were detected in liver explant tissue and plasma samples of the cases. Epstein-Barr virus and varicella zoster virus infection with pathognomonic clinical symptomatology preceded liver failure in two respective cases. AAV2 was detected in one-third of control livers. Excessive activation of monocyte pathways was detected in liver explants from cases compared with controls. Remarkably, this signature was comparable for AAV2, adenoviruses, and/or herpesviruses-positive transplant cases. Our multi-omic findings suggest a common transcriptomic profile, with an upregulation of monocyte pathways in the presented transplanted cases, which had similar severe clinical outcomes. In the cohort presented, AAV2 was not exclusively associated with acute liver failure, suggesting that other processes may have contributed to a uniform cascade of irreversible pathology.

**IMPORTANCE:**

Since the appearance of the cluster of pediatric hepatitis of unknown origin in 2022, several groups have reported an association of adenoviruses and AAV2 in a high number of cases in contrast to controls. The adenoviruses detected were heterogeneous in both species—adenovirus C and F—and sequences. The mechanisms of disease that accounts for fulminant liver failure, occurring in 5% of pediatric hepatitis cases, remain incompletely described. The current study adds to previous data by including pediatric acute liver failure cases during the upsurge, enabling the analyses of inflammation expression profiles in cases with different viruses in relation to pediatric controls. This led to the discovery of transcriptome upregulation of monocyte pathways in liver explants from the cases. This inflammatory transcriptomic signature was comparable for AAV2, adenoviruses, and/or herpesviruses-positive transplant cases.

## INTRODUCTION

During spring 2022, 35 countries in five World Health Organization (WHO) regions reported a cluster of over 1,000 cases of severe childhood hepatitis without a known cause, with 5% progressing to acute liver failure (1). In the Netherlands, a documented cohort of 17 children was hospitalized with acute severe hepatitis, as defined by the WHO. In total, five children were treated for acute liver failure at the national pediatric transplant center. The clinical characteristics of these children have been described before (2).

Last year, several groups independently reported high levels of adeno-associated virus 2 (AAV2) genome in 93%–100% of these pediatric hepatitis cases in contrast to controls (3–5). Their corresponding liver tissue analyses implied extensive immune responses, whereas viral AAV2 particles and proteins remained undetected (4, 6–8). The mechanisms of disease that accounts for fulminant liver failure in these patients remain incompletely described.

We aimed to analyze the available liver tissue and plasma samples to further elucidate the mechanisms involved in the fulminant decay of liver function in previously healthy children. This quest aims at the identification of immune transcriptomic profiles and potential targetable groups of gene transcripts present in these affected children. Using targeted transcriptomics and metagenomic analysis, we performed an in-depth molecular analysis of the mechanism of pediatric acute liver failure.

## RESULTS

**Fig 1:**
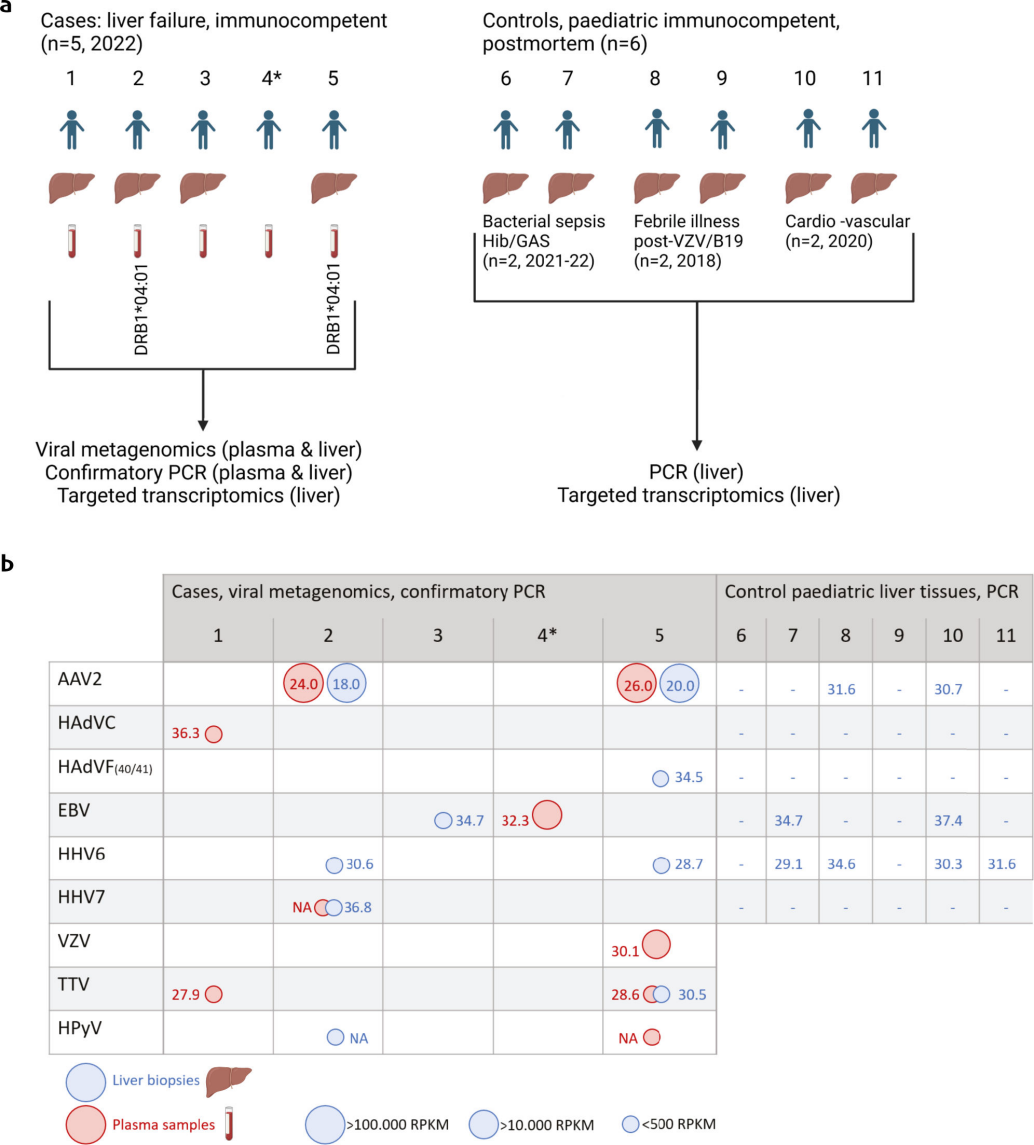
(a) Study design. Cases of non-A-E hepatitis (*n* = 5) and non-hepatitis controls (*n* = 6). Explanted liver biopsies and plasma samples from cases were subjected to metagenomic sequencing analyses. Controls were tested by PCR for metagenomic findings in the cases. Liver tissue samples from both cases and controls were subjected to transcriptomics. HLA DRB1*04:01 positivity is indicated. *Not transplanted, no liver explant available. Hib, *Haemophilus influenzae* b; GAS, group A streptococcus; VZV, varicella zoster virus; B19, human parvovirus B19. (b) Viral metagenomic detection of viruses in pediatric cases of acute liver failure. Numbers correspond to Ct values of confirmatory PCRs. Circle sizes are representative of the number of sequence reads normalized per virus genome size (RPKM, reads per kilobase genome per million). − , not detected; HHV, human herpes virus; VZV, varicella zoster virus; TTV, torque teno virus; HPyV, human polyomavirus. Created using Biorender.com.

All pediatric cases presenting with hepatitis A–E negative acute liver failure in the Dutch national reference center for liver transplantation during the spring 2022 outbreak were included. This led to the enrollment of a total of five cases admitted from weeks 11–17, representing an unprecedented peak in the incidence of pediatric acute liver failure, which has averaged approximately two national cases/year over the last two decades. The enrolled cases had a median age of 3 years (range: 11 months to 8 years) and were all immunocompetent and previously healthy. Cases 2–5 presented with jaundice and vomiting; case 1 (11-month-old) presented with vomiting, diarrhea, and anorexia. Case 1 was the most clinically fulminant, presenting with circulatory insufficiency and requiring stress dose hydrocortisone. None of the other cases had received steroids. Upon admission at the pediatric intensive care unit, a protocolized comprehensive work-up was performed to identify the causes of liver failure, including medical history, metabolic screening, vascular liver analysis, genetic screening, virology panels, and toxicology screening, including paracetamol (2). Three of the cases were SARS-CoV-2 IgG seropositive. Four of five patients underwent a successful emergency living donor protocol procedure within 6 days (cases 1 and 2) to 2 weeks (cases 3 and 5) after onset of vomiting/jaundice. One patient recovered spontaneously (case 4). Histology of the explanted liver tissues showed hepatocytic decay without fibrosis in all cases, with ballooning and lymphoid inflammatory infiltrate in all but less prominently in case 1. Plasma and explanted liver tissue samples of the cases were available for viral metagenomic and transcriptomic analyses (Fig. 1).

As controls, postmortem liver biopsies from a total of six age-matched immunocompetent children in the LUMC Biobank Infectious Diseases were available. The median age of these controls was 7 years (range: 8 months to 17 years), and all had died suddenly in non-hospitalized settings. The controls were suffering from acute bacterial sepsis, cardiovascular death, and acute febrile illness linked to parvovirus B19 and post-varicella myocarditis, respectively (Fig. 1a). The two controls with sepsis (6 and 7) were sampled contemporaneously with the liver failure cases. Control 10 was SARS-CoV-2 IgG-positive, the serostatus of controls 6, 7, and 11 was unavailable, and controls 8 and 9 were transplanted pre-pandemic (2018). Main clinical characteristics of pediatric cases and controls are listed in Table S1.

### Viral metagenomics reveals multiple viruses and PCR AAV2-positive controls

Of the pediatric cases presented with hepatitis A-E-negative acute liver failure, explanted liver tissue and paired plasma samples were analyzed using a sensitive metagenomic protocol targeting vertebrate viruses including novel ones (9–11). Metagenomic and confirmatory PCR results are shown in Fig. 1b; read counts and coverage maps are depicted in Fig. S1. By metagenomics and PCR, AAV2 was detected in high loads in two of the five cases (cases 2 and 5), in conjunction with other viruses. In case 5, AAV2 detection coincided with varicella zoster virus detection, with concurrent pathognomonic symptomatology preceding the acute liver failure. AAV2 detection coincided with the detection of up to five other viruses, whereas AAV2-negative liver tissue samples were characterized by the detection of a single, or in one case, two other viruses. The two AAV2-positive patients were genotyped positive for the class II HLA-DRB1*04:01 allele (cases 2 and 5, Fig. 1a), which has been associated with hepatitis A-E-negative AAV2 cases (3). In the AAV2-negative cases, Epstein-Barr virus (EBV; cases 3 and 4) and human adenoviruses type C (case 1) were detected. EBV detection in case 4 supported serological evidence of acute EBV infection (IgM and IgG VCA positive, IgG EBNA negative). Low load polyomaviruses were detected in cases 2 and 5. No confirmatory PCR was available, but it has been described that 4% of the Dutch blood donors are HPyV DNA-positive (12). Of note, although liver tissues were HAdV-negative for cases 2 and 4, low HAdV loads were detected in plasma (case 2) and respiratory swabs (case 4) in combination with negative feces samples (2).

The control liver tissues, from age-matched immunocompetent children with sudden and initially unexplained deaths in 2022 and pre-Covid-19 pandemic years (see above), were screened with directed PCRs for targets detected by metagenomic sequencing in the cases. The most common finding by PCR in controls was HHV6, in four of six controls. AAV2 was detected in two pediatric controls, one with post-VZV febrile illness (control 8, 2018) and one with sudden onset cardio-vascular death (control 10, 2020).

### Monocytes predominating inflammation profile among the cases

**Fig 2:**
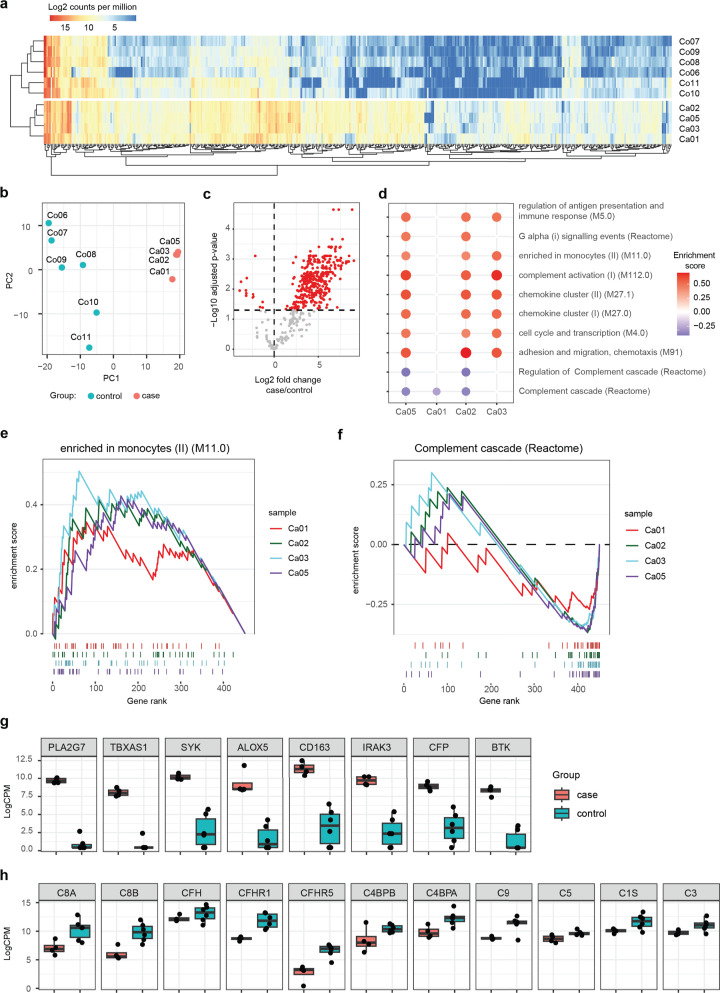
Transcriptomics results of liver samples from transplanted patients and controls. (**a**) Heatmap of the expressions of inflammation gene transcripts targeted, in cases (*n* = 4) and controls (*n* = 6), as log_2_ counts per million (Table S2). (**b**) Principal component analyses of gene transcripts in cases and controls. (**c**) Volcano plot showing differentially expressed genes, with all genes included. (d–f) Single sample gene set enrichment analysis (ssGSEA), comparing cases against the average of controls. Reactome and blood transcriptional modules (14) were included as pathways. Only pathways with statistically significant enrichment (*P*-adjusted < 0.05) in at least two cases are shown. Size and color indicate direction and magnitude per pathway and case. Enrichment scores are shown for two example pathways per case. Colored lines below depict the rank, based on fold change relative to controls, of all genes present in the pathway. Highest-ranking genes indicate an increase relative to controls and are shown on the left, and lowest-ranking genes indicate the greatest decrease relative to controls and are shown on the right. (**g**) Monocyte/macrophage-derived pro-inflammatory core (leading edge) genes in cases. (h) Decreased expression of complement pathways leading edge genes in cases compared with controls. Boxplots center line, median; box limits, upper and lower quartiles; whiskers, 1.5× interquartile range; points, outliers.

Transcriptomic sequencing was conducted to better understand the immunological response in the liver of pediatric acute liver failure cases (13). Available liver tissue samples from cases and controls were subjected to transcriptomic inflammatory profiling. We used a validated amplicon-based approach for increased detection of transcripts in low input samples, targeting a panel of >600 inflammatory gene transcripts (of which 452 were detected). The results of the transcriptomic profiling are shown in Fig. 2. Expression profiles of inflammation gene transcripts in cases showed transcriptomic inflammatory signatures very different from those of the controls, with upregulation of macrophage/monocyte pathways (Fig. 2a). Principle component and hierarchical clustering analyses based on all genes showed a clear clustering of profiles of cases compared with controls (Fig. 2a and b). The majority of gene transcripts were increased in cases over controls, although transcription of several genes and, in particular, complement genes that are produced in the liver were decreased in cases (Fig. 2c). Full tables of differentially expressed genes can be found in Table S2. Enrichment analysis of the analyzed genes showed that several transcriptional modules were significantly enriched among these immunological genes (Fig. 2d), including “regulation of antigen presentation and immune responses (M5.0)” (including genes *TLR2, CCR7*, *CD4*); “enriched in monocytes (II) (M11.0)” (including genes *CD163*, *PLA2G7*, *ALOX5*, *CCR1*) (Fig. 2e); and “chemokine clusters (I &II, M27.0 & M27.1)” (including genes *CXCL6* and *CCL5*). In contrast, complement activation (C1/7) was significantly reduced in cases at a gene and pathway level (Fig. 2f), indicative of reduced synthesis by hepatocytes. Although all cases were clearly distinct from controls, case 1 had a somewhat different enrichment profile; he had received hydrocortisone treatment due to the fulminant clinical course, in contrast to other cases, and presented relatively later in the course of the liver disease. Within the explanted cases, core (leading edge) gene expression patterns were comparable, implying a shared transcriptomic inflammatory signature, although different viruses were detected in their livers (Fig. 2g and h).

### Proteomic analysis of explanted livers

To validate the transcriptomic data at a protein level, we used the targeted Olink platform to analyze liver tissue lysates. Samples of three cases and four controls could be homogenized, passed quality controls, and analyzed. Of the 92 targeted proteins, 61 proteins were detectable, of which 27 could be directly linked to measured gene transcripts. Of these, 16 had a Pearson rho > 0.5 when correlating gene transcript and protein levels (Fig. S2a). These included chemokines and cytokines involved in monocyte/macrophage activation such as CCL2 (rho = 0.79, *P* = 0.033) and IL-18 (rho = 0.70, *P* = 0.082) but also TNFRSF9/CD137 (rho = 0.97, *P* = 0.00033), which is a marker of activated T cells. Of the 61 detectable proteins, 19 were increased in the cases and one (VEGF-A) was increased in controls with uncorrected *P*-value < 0.05 (Fig. S2b). Reduced levels of VEGF-A may be due to the destruction of hepatocytes, which are producers of VEGF-A (15). The increased proteins included 14 chemokines and cytokines, in line with the upregulated pathway of chemokine clusters I and II, as observed by pathway analysis of the transcriptomic data.

### Integration with other transcriptomic data sets of acute liver failure

**Fig 3:**
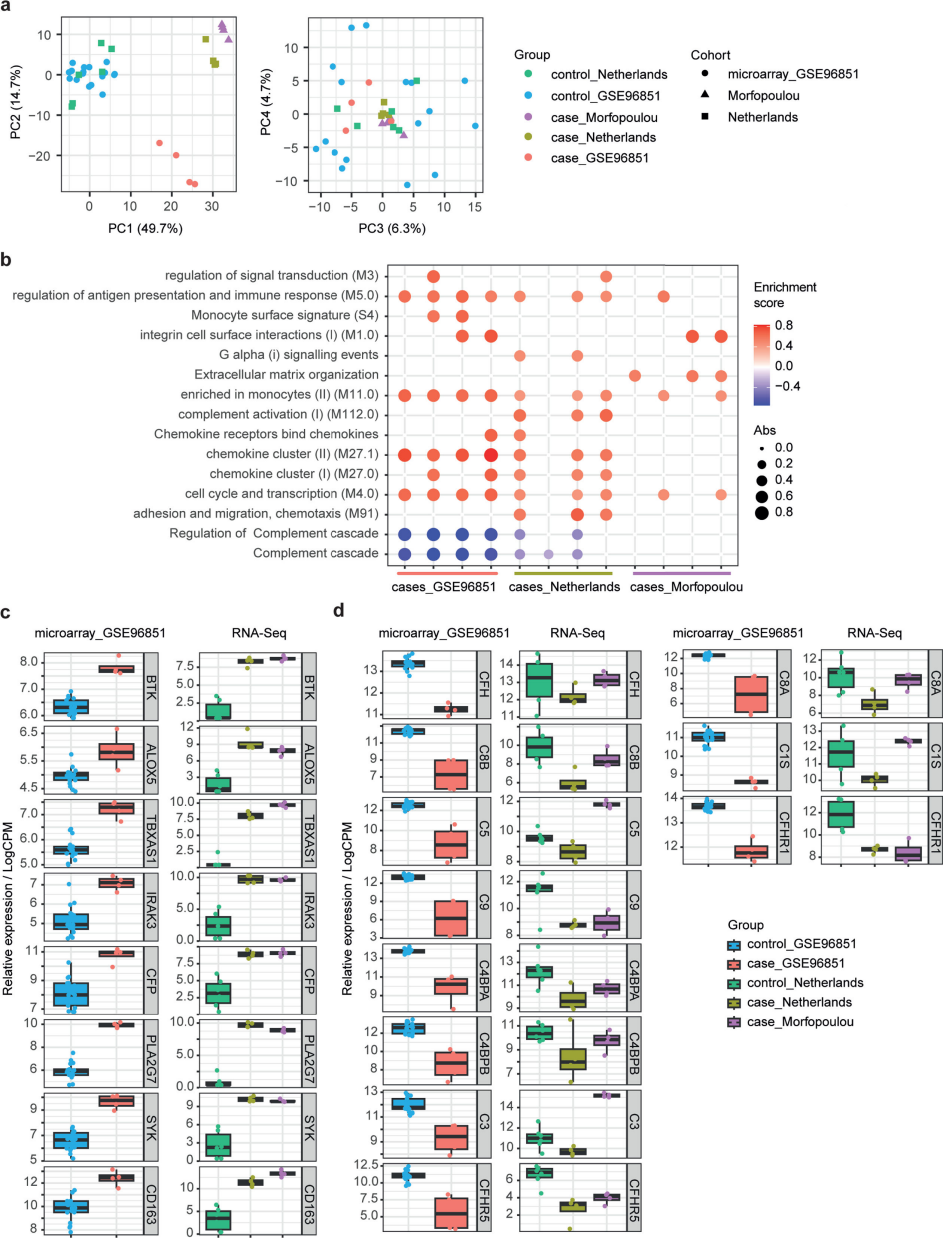
Transcriptomics integration of liver samples with published cohorts. (**a**) Principal component analyses of z-scale normalized gene expression levels in cases and controls (PC1/2 on left and PC3/4 on right). This includes 17 controls from study GSE96851 (blue), six controls from this study (green), four HBV-associated acute liver failure cases from study GSE96851 (red), four pediatric acute liver failure cases from Morfopoulou et al. (purple) and four pediatric acute liver failure cases from this study (olive). (**b**) Single sample gene set enrichment analysis (ssGSEA), comparing GSE96851 cases against the average of GSE96851 controls, and cases from this study and Morfopoulou et al. against our cases. Reactome and blood transcriptional modules (14) were included as pathways. Only pathways with statistically significant enrichment (*P*-adjusted < 0.05) in at least two cases are shown. Size and color indicate direction and magnitude per pathway and case. (c and d) Boxplots showing leading edge genes expression levels (either normalized expression levels from microarray or log count per million from RNA-Sequencing) from (c) the “enriched in monocytes” pathways and (**d**) the “complement cascade.” Boxplots center line, median; box limits, upper and lower quartiles; whiskers, 1.5× interquartile range; points, outliers.

We then wanted to understand how similar our cases were to a previously described RNA-seq data set of pediatric acute liver failure cases by Morfopolou et al. (4) and gene expression microarray data set of HBV-associated acute liver failure from Chen et al. (16) Data were normalized at a gene transcript level based on control samples, taking into account only the genes included in our targeted approach. Our cases clustered together with those from Morfopoulou and separately from the controls, based on principal component 1, and the HBV-associated acute liver failure cases, based on principal component 2 (Fig. 3a). Components 3 and 4 explained only 6.3% and 4.7% of the variation and did not differentiate between the groups. HBV-associated acute liver failure cases clustered separately, and this could be due to biological or technical differences in microarray and RNA-seq. We therefore performed ssGSEA analysis, including all cases as described above, in which ranks rather than expression values are used (Fig. 3b). The pathway “enriched in monocytes (II) (M11.0)” was significantly upregulated in 9 of 12 cases across all studies, as was the pathway “cell cycle and transcription (M4.0).” The monocyte genes were also clearly enriched in cases across all studies (Fig. 3c), confirming our findings in two additional cohorts and identifying key inflammatory markers associated with acute liver failure. Surprisingly, and in contrast to the finding from principal component analysis, our cases more resembled at a pathway level the HBV-associated acute liver failure cases than the cases described in Morfopoulou et al. (Fig. 3c). For example, the “complement cascade” pathway, as well as complement genes, was reduced in our cases and those from Chen et al., but not those from Morfopoulou et al. (Fig. 3d). This might indicate differences in stage of disease at the time of transplant, but drawing firm conclusions from such integrations is impossible due to the potential of batch effects confounding biological differences.

## DISCUSSION

This exploratory study aimed to elucidate the different pathways involved in the process of pediatric acute liver failure, during the 2022 upsurge. We provide complementary metagenomic and transcriptomic data, suggesting that a common monocyte/macrophage inflammatory transcriptomic and proteomic profile was associated with the transplanted cases, although different viruses were detected in their livers. AAV2 was detected in two cases in conjunction with several herpesviruses including VZV and adenovirus type F. AAV2-negative cases were EBV and adenovirus type C positive. The finding of multiple viruses is in line with reports that have not excluded—for instance, EBV-positive hepatitis cases after metagenomic detection (5). It has been hypothesized that concomitant detection of multiple helper viruses, such as HAdV and HHV6B, in the cases may have resulted in higher AAV2 loads (8), which would be in line with our data. Importantly, AAV2 detection was not limited to cases: two controls with histologically normal liver tissues sampled in 2018 and 2020 were positive for AAV2, with lower loads. Strikingly, the inflammatory signature was comparable for AAV2, adenoviruses, and/or herpesviruses positive transplant cases, and the cases found here showed a similar profile to previously described cases by Morfopoulou et al. These findings suggest a common pathway of immunopathology, potentially linked to different viruses circulating during the cluster, with similar severe clinical outcomes. It has been suggested that a proportion of cases might simply be due to the increased circulation of various infectious agents observed after the lockdown rather than to a single virus (8).

Given the upregulated transcriptomic markers in the cases, hepatic macrophages (Kupffer cells) are likely involved not only in triggering hepatocytic cell death but also in protective signaling pathways and assistance in T cell priming (17, 18). Multiple immune genes relevant to monocyte/macrophage function showed increased transcription in the cases, suggesting the induction of not just pro-inflammatory but also regulatory responses. For example, increased transcription of *CD163* in these liver samples agrees with earlier reports showing that sCD163 could be a plasma biomarker for Kuppfer cell activation (19). *ALOX5* encodes the enzyme Arachidonate 5-lipoxygenase that is required for the synthesis of leukotrienes, such as LTB_4_ (20), but also leads to the generation of specialized pro-resolving mediators (SPMs), lipids that are required for the resolution of inflammation. In addition, *TGM2* and *TGFB* were among the upregulated genes in cases (Table S2), suggestive of efferocytosis, the process of clearing apoptotic cells, alternative macrophage polarization (21–23), and tissue repair. Other genes that might be involved in upregulation are also induced. Phospholipase A2 group VII (PLA2G7) is an enzyme involved in phospholipid metabolism. It was recently shown that PLA2G7high macrophages found within tumors are highly immunosuppressive (24). Interleukin 1 receptor-associated kinase 3 (*IRAK3)* is an important negative regulator of TLR signaling, impairing NF-kB activity (25). Several kinases important for myeloid activation were found upregulated. *BTK* encodes a non-receptor kinase (Bruton’s tyrosine kinase) that is important for myeloid cell activation and recruitment, as its inhibition in tissue macrophages impairs chemokine secretion by reducing NF-kB activity and Akt signaling, whereas it also reduces monocyte chemotaxis (26). Likewise, spleen tyrosine kinase (*Syk*) is an important kinase that mediates TLR signaling and also Fc receptor-dependent signaling (27). To further elucidate these associations and determine whether blood-based transcriptomic biomarkers related to monocyte/macrophage activation could be used to predict clinical course, further investigations will be required. Comparing transcriptomic data with protein levels using Olink further confirmed the inflammatory profile observed in explanted liver cases.

In this study, the small number of cases limited the statistical power to assess potential associations with the different viruses detected. Although all Dutch cases treated during the outbreak were enrolled in this study, the number of cases remained low due to the focus on the most severe outcome: liver failure. Second, no pediatric control livers with “classical” viral hepatitis (A–E) could be included due to unavailability in the biobank related to the low incidence among children. Due to the sudden onset of death of the children, liver biopsies were used as controls in this study, AST/ALT levels were not available for all controls, and control livers were not assessed by viral metagenomics. No conclusions can be drawn on the causality of the different viruses and associations found in relation to acute liver failure in the current exploratory study.

This study adds to earlier studies using genomics during the outbreak by focusing on the pathogenesis of the recent pediatric hepatitis upsurge. The inclusion of liver tissues of immunocompetent pediatric controls resulted in a relatively high proportion of AAV2-positive non-liver failure tissues. Although some investigations included only cases that had previously tested positive for HAdV, the present study included acute liver failure cases, irrespective of HadV finding, and thus provided the opportunity to profile AdV, AAV2, and herpesvirus-positive cases.

Additional *in vitro* and *in vivo* studies are needed to address the possible pathogenicity of AAV2 in combination with different viruses and the timing of viral replication in relation to the inflammatory response in naïve and non-naïve individuals. Several pro-inflammatory cytokines overexpressed in our cases are targets of currently available anti-inflammatory drugs, including CD80, IL18, IL6, IL33, IL15, IL1, and IL12 (Table S2), and their role may be studied in more cases to map a potential benefit in the prevention of progression of hepatitis to acute liver failure, thus averting a liver transplant in a previously healthy child. International databases and sample repositories should be set up in collaboration, storing processed transcriptomic and other multi-omics data and enabling access to data on controls. This will enable enlarged statistical power when performing a multi-omics analysis to unravel the etiopathogenesis of an emerging infectious disease.

## MATERIALS AND METHODS

### Patients

All pediatric cases presented with hepatitis A–E-negative acute liver failure (INR ≥ 2.0, transaminases elevated >1,000 U/mL, and some degree of hepatic encephalopathy) at the Dutch national reference center for liver transplantation in the spring of 2022 were included in the study. Standardized work-up for the detection of known liver failure causalities was performed; metabolic, toxic, and inborn liver errors were ruled out during admission. Routine virological analyses were performed for hepatotropic causes, as described by Lexmond et al. (2).

Postmortem liver biopsies from a total of six age-matched immunocompetent children were available as control samples from the LUMC Biobank Infectious Diseases. The controls died suddenly and were initially unexplained in non-hospital settings. Post-mortem evaluation revealed acute bacterial sepsis, cardio-vascular sudden death, and febrile illness linked to parvovirus B19 and post-varicella myocarditis, respectively. The two controls with sepsis were sampled contemporaneously with the liver failure cases. Liver function data were available for two of the controls: control 10, with a cardio-vascular cause of death, had, post-resuscitation, AST/ALT levels of <500 IU/L, and control 6, who died of Hib sepsis/meningitis, had a multi-organ failure with AST/ALT levels of >1,000 IU/L. Postmortem microscopic examination of the controls with unavailable AST/ALT levels did not reveal signs of hepatitis or cellular decay. HLA typing data were not available for controls. Liver tissue and plasma samples were stored at −80°C.

### Viral metagenomics

Viral metagenomic analyses of explant liver and plasma samples were performed using a sensitive probe capture hybridization method targeting all known human and vertebrate viruses and suited for virus discovery as described before (9, 28, 29). The limit of detection has been determined as 10–100 c/ml for ssRNA and dsDNA viruses. All samples were spiked with internal controls prior to nucleic acid extraction, consisting of low loads of equine arteritis virus and phocid herpes virus. In short, nucleic acids were extracted directly from plasma samples using the MagNApure 96 DNA and Viral NA Small volume extraction kit (Roche, Basel, Switzerland), liver biopsy specimens were pre-treated with vortexing 5 min 2,200 rpm in 1.25 ml STAR-buffer and Precellys beads, followed by centrifugation for 1 min at 14,000 rpm. DNA and RNA library preparation was performed using NEBNext Ultra II Directional RNA Library prep kit (New England Biolabs, Ipswich, MA, USA) for Illumina according to the manufacturers protocol. As in-house adaptations all poly A mRNA capture isolation, rRNA depletion, and DNase treatment steps were omitted to enable simultaneous detection of both DNA and RNA in a single tube per sample. Negative controls receiving the identical extraction and library preparation method were used in each hybridization pool. For the viral target enrichment, overnight hybridization (30) took place using the SeqCap EZ HyperCap (Roche) according to the manufacturer’s protocol, after which 150 bp sequencing using the NovaSeq6000 sequencing system (Illumina, San Diego, CA, USA) was performed. This viral metagenomics enrichment protocol has been validated for clinical use (10, 28, 31, 32) in one of the study centers.

Virus genome assembly, alignment, and taxonomic classification results were obtained using GenomeDetective (v2.40) (33) and Centrifuge (v1.0.3-beta) (34). GenomeDetective first performs quality filtering and trimming, followed by assembly of extracted viral reads and taxonomic classification. Centrifuge is a k-mer based tool for taxonomic classification, lowest common ancestor settings were applied. Additional analysis was performed by mapping all reads separately using Bowtie2 (v2.1.0) (35) for AAV-2 NC_001401.2. Samples with a high number of reads being classified by Centrifuge and that were not found by GenomeDetective (33), were additionally mapped using Bowtie 2 (v2.1.0) (35) for EBV NC_009334.1, HAdVC NC_001405.1, HHV7 NC_001716.2, TTV10 NC_014076.1 and TTV28 NC_014073.1. The resulting sequence alignments were converted to BAM format, sorted, indexed using SAMtools (v1.14) (36). Fasta files were created from the BAM files using every part of the genome that was covered >1×, the fasta files were then mapped and visualized using the annotated genome aligner AGA (37), a subtool of GenomeDetective (33).

### PCRs

Metagenomic findings were confirmed by PCR, preceded by repeated nucleic acids extraction. Controls were tested by PCR for metagenomic findings in the cases. An overview of primer and probe sequences is listed in Table S3. AAV2 PCR was developed by the Dutch National Institute for Public Health and the Environment; primers and probes targeted the NSP and VP1 regions (38). RNA was extracted from 190 ul sample with the addition of 10 ul internal control, phocine distemper virus (PDV), using the NucliSense EasyMag (bioMérieux, Lyon, France). qPCR was performed in a total reaction volume of 20 µl using 5 µl RNA, 1× TaqMan Fast Virus 1-Step Master Mix (Applied Biosystems, Foster City, CA, USA), 500 nM forward and reverse primers for AAV2 VP1 or NSP, 250 nM AAV2 VP1 or NSP probes, 300 nM forward and reverse primers for PDV, 100 nM probe for PDV and DNase/RNase free water (Sigma). Reactions were run on an ABI7500 using the profile of 15 min 50°C, 3 min 95°C, followed by 50 cycles of 10 s 95°C and 30 s 55°C. PCRs targeting other viral pathogens were performed as described previously (28, 39).

### Transcriptomics sequencing

Targeted transcriptomics for low input liver biopsies was performed using the AmpliSeq for Illumina RNA Inflammation Response Research Panel (Illumina, Inc.), consisting of 724 amplicons/primer pairs covering 683 targets genes involved in key pathways of the inflammatory response. For each sample, 100 ng of total RNA was used for the library preparation, followed by sequencing on the Novaseq6000 according to manufacturer's protocols.

Bioinformatic analysis was performed using the RNA-seq pipeline of BioWDL (https://biowdl.github.io/RNA-seq/v5.0.0) based on GRCh38 and STAR aligner (v2.7.5a). Downstream analysis scripts used for immunological analysis are available: https://github.com/spjochems/pALF. For comparison of cases and controls, *t*-tests were used on log_2_ counts per million normalized reads, with Benjamini-Hochberg correction for multiple testing. For single sample gene set enrichment analysis, the “fgsea” package (v1.24.0) (40) was used to assess enrichment for genes related to blood transcriptional modules and Reactome pathways (14). Leading-edge genes, or *c*ore genes that account for the gene set's enrichment signal, were defined as those that appear in the ranked list before the point at which the running sum of the rank metric reaches its maximum deviation from zero.

### Integration with other transcriptomic datasets

HBV-associated acute liver failure data were downloaded from GSE96851. For gene transcripts with multiple probes, a median expression was calculated per gene. Multiple technical replicates were present per cases, and a median expression value was calculated per sample. Liver angioma and control samples were present in the data set and are all labeled as control, as they showed a similar profile. Read counts from Morfopoulou et al. (4) were provided by the authors and log counts per million were calculated per sample. Only genes present in the current dataset were retained for all data sets. To normalize between data sets, a modified z-score was calculated per gene and per dataset where each data point had the mean of the controls from the dataset subtracted, followed by dividing by the standard deviation of all samples in that data set. The Morfopoulou was added to the current data set given the similar profile prior to correction.

### Olink analysis

For proteomic analysis, a needle point of liver tissue for all patients was collected in 10 µL of lysis buffer (Bio-Plex Cell Lysis Kit #171304011, containing serine protease inhibitor phenylmethanesulfonyl fluoride, Cell Signaling Technology). We then performed 15 sonicator cycles, consisting of 10 seconds 45 kHz sonication + 1 min on ice, and froze down at −80°C. On day of analysis tissue samples were resuspended in lysis buffer, centrifuged at 1000xg 5 min to separate supernatant. Protein analysis was performed using the Olink Target 96 Inflammation panel and was measured on a Q100 Signature (Olink) machine at Genomescan, Leiden. The NPX Signature software was used for QC analyses and to generate normalized protein expression (NPX) values at a log_2_ scale. Samples of 3 cases and 4 controls passed the quality control and were analysed. A control sample consisting of lysis buffer was included to define background values per protein, and proteins for which the average expression in cases or controls was >1 NPX increased relative to background were retained. Comparison of expression between cases and controls was performed using *t*-test per protein. Correlation with transcriptomic data was done using Pearson correlation test.

## Data Availability

Full AAV2 genome sequences were obtained and uploaded to GenBank (OR119757.1, OR129877.1, OR129878.1, and OR129879.1). Other sequencing data are available upon request given the ethical regulatory restrictions linked to the consent.

## SUPPLEMENTAL MATERIAL

Fig. S1 mbio.03913-24-s0001.docx Sequence read coverage plots of main virus findings and of AAV2 genome from pediatric cases of acute liver failure.

Figs S2 mbio.03913-24-s0002.pdf Protein expression as measured in liver tissue lysates of pediatric cases and controls.

Table S1 mbio.03913-24-s0003.docx Main clinical characteristics of paediatric acute liver failure cases and pediatric controls.

Table S2 mbio.03913-24-s0004.xlsx Overexpressed gene transcripts (as visualized in the volcano plot in Fig. 2).

Table S3 mbio.03913-24-s0005.xlsx PCR primers and probe sequences.

ASM does not own the copyrights to Supplemental Material that may be linked to, or accessed through, an article. The authors have granted ASM a non-exclusive, world-wide license to publish the Supplemental Material files. Please contact the corresponding author directly for reuse.
