# Supplementary material for: Metagenomic and transcriptomic investigation of pediatric acute liver failure cases reveals a common pathway predominated by monocytes
Source: mBio. 2025 Mar 18;16(4):e03913-24. doi: 10.1128/mbio.03913-24 (PMC11980388; doi:10.1128/mbio.03913-24)
Supplement: Fig. S1 — Sequence read coverage plots of main virus findings and of AAV2 genome from pediatric cases of acute liver failure. [file mbio.03913-24-s0001.docx]

**Supplementary Fig 1. Sequence read coverage plots of main virus findings and of AAV2 genome from paediatric cases of acute liver failure.**

**a**

|  | **Plasma** findings - read counts and coverages | **Liver** findings - read counts and coverages |
| --- | --- | --- |
| Patient 1 | Centrifuge^1^:  HAdVC 459 reads, PCR Ct-value 36.3  Bowtie2/AgaAligner 0.4% coverage  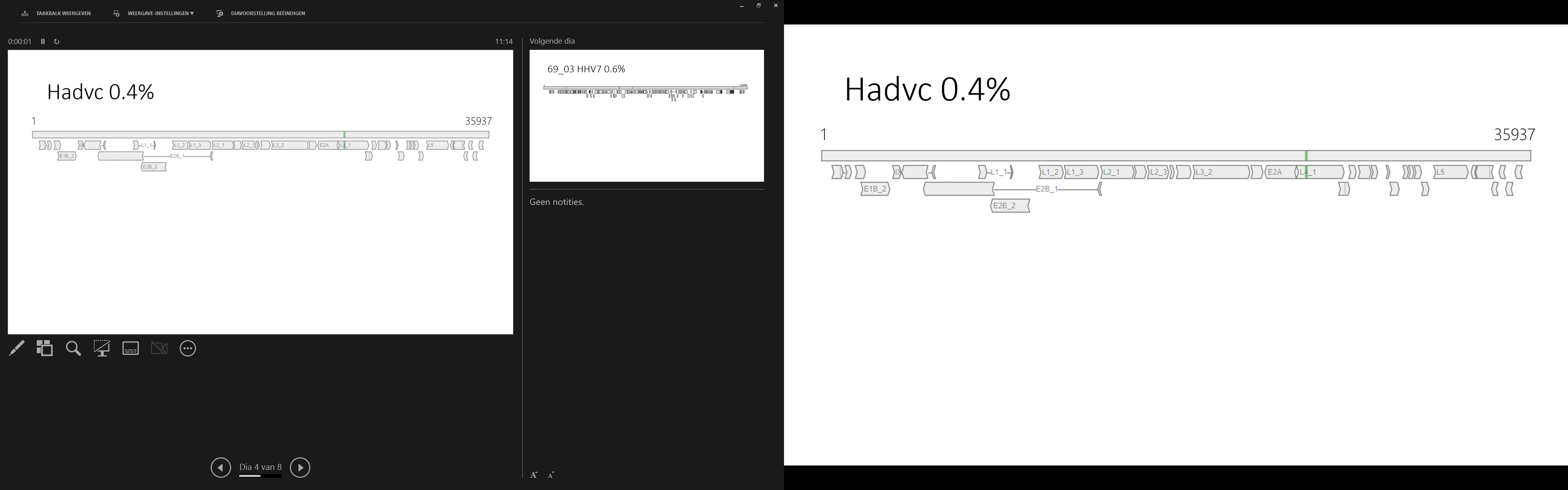  Alphatorque, including TTV28 169 reads,  PCR Ct-value 27.9  Bowtie2/AgaAligner TTV28 16.6% coverage  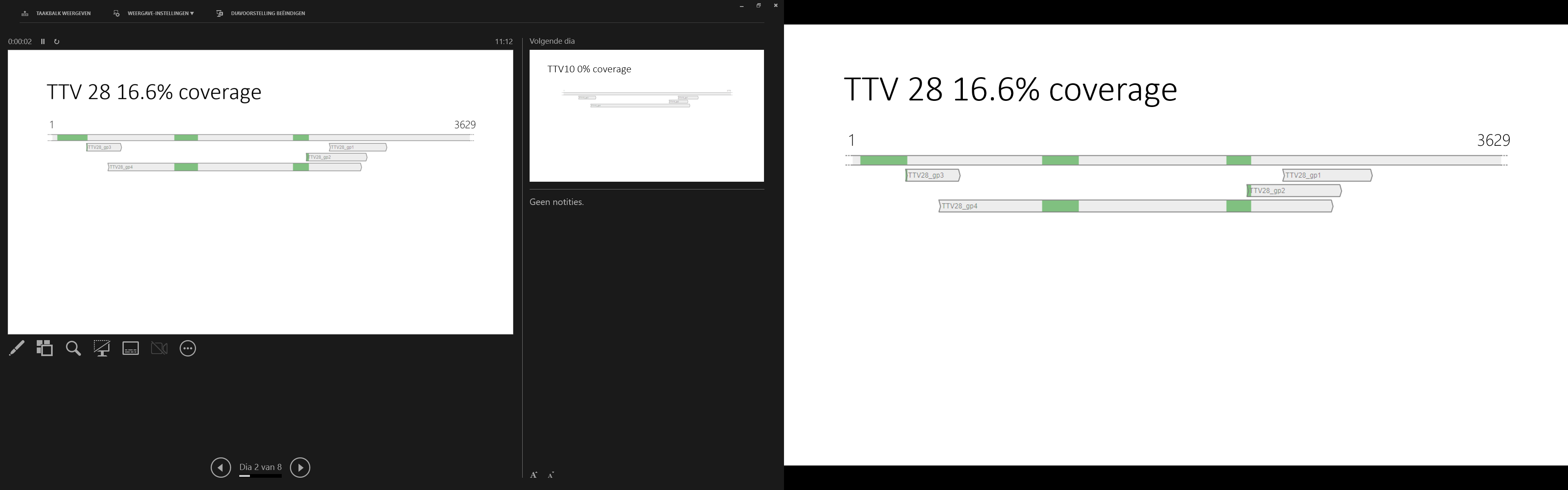 | None |
| Patient 2 | GenomeDetective:  AAV-2 994,335 reads, 100% coverage, PCR Ct-value 24  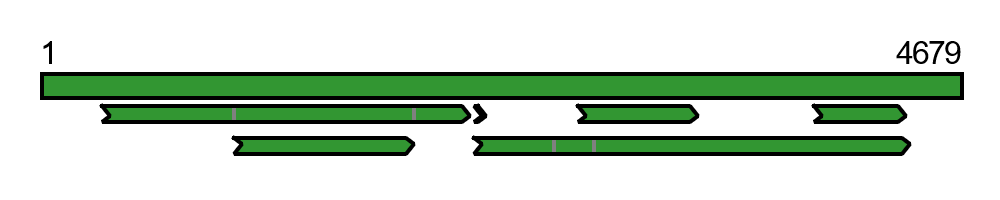  Centrifuge^1^:  HHV7 100 reads, PCR NA^2^  Bowtie2/AgaAligner 0.6% coverage  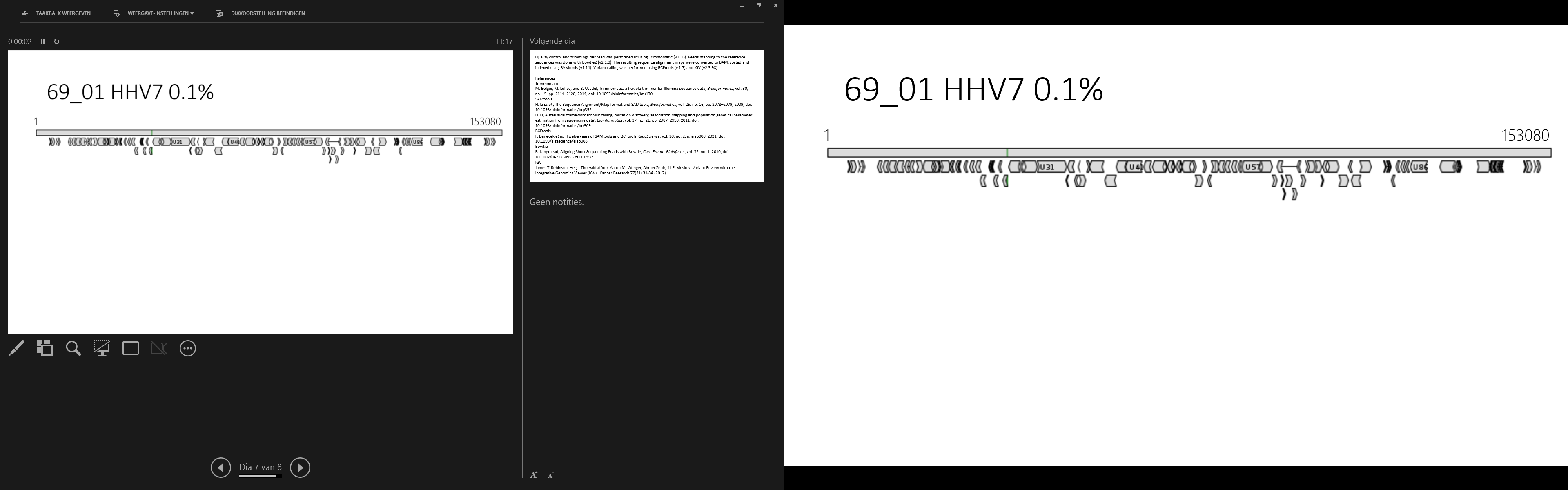 | Genome Detective:  AAV-2 1,488,305 reads, 99.9% coverage  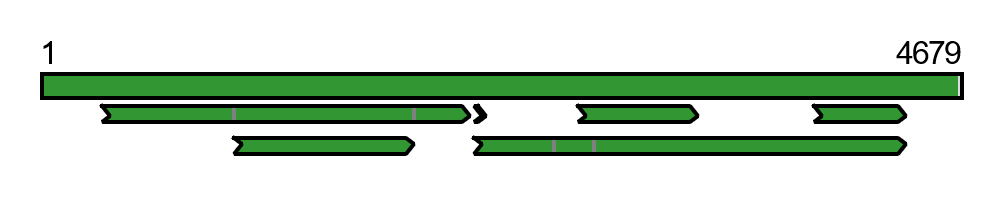  HHV-6 6,286 reads, 13% coverage, PCR Ct-value 30.6 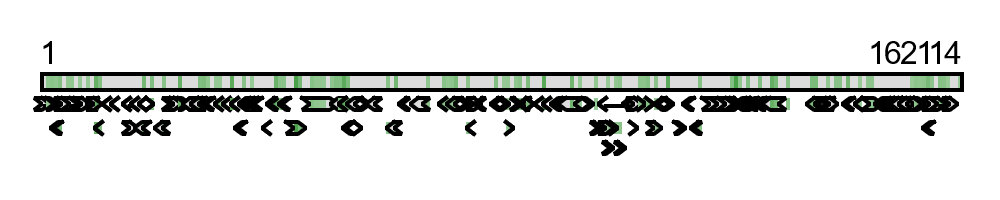  HHV7 252 reads, 1.4% coverage, PCR Ct-value 36.8  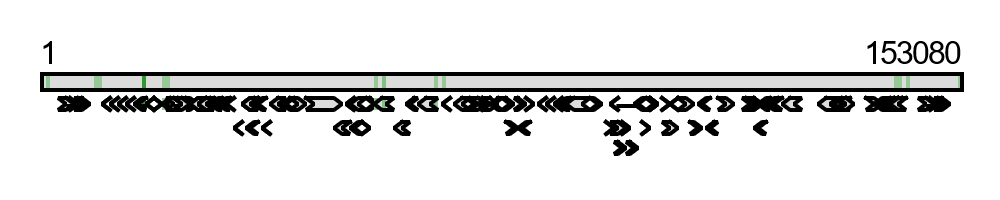  HPyV 292 reads, 12.2% coverage  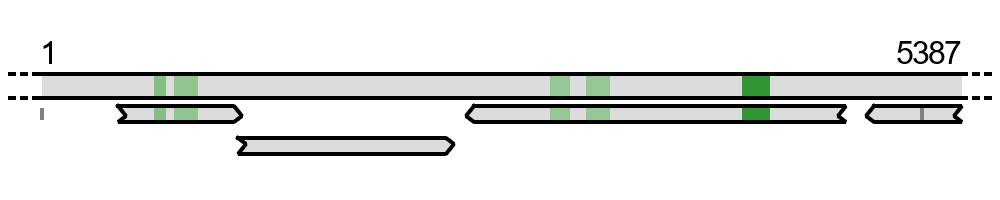 |
| Patient 3 | None | Centrifuge^1^:  EBV 173 reads, PCR Ct-value 34.7  Bowtie2/AgaAligner 0.3% coverage  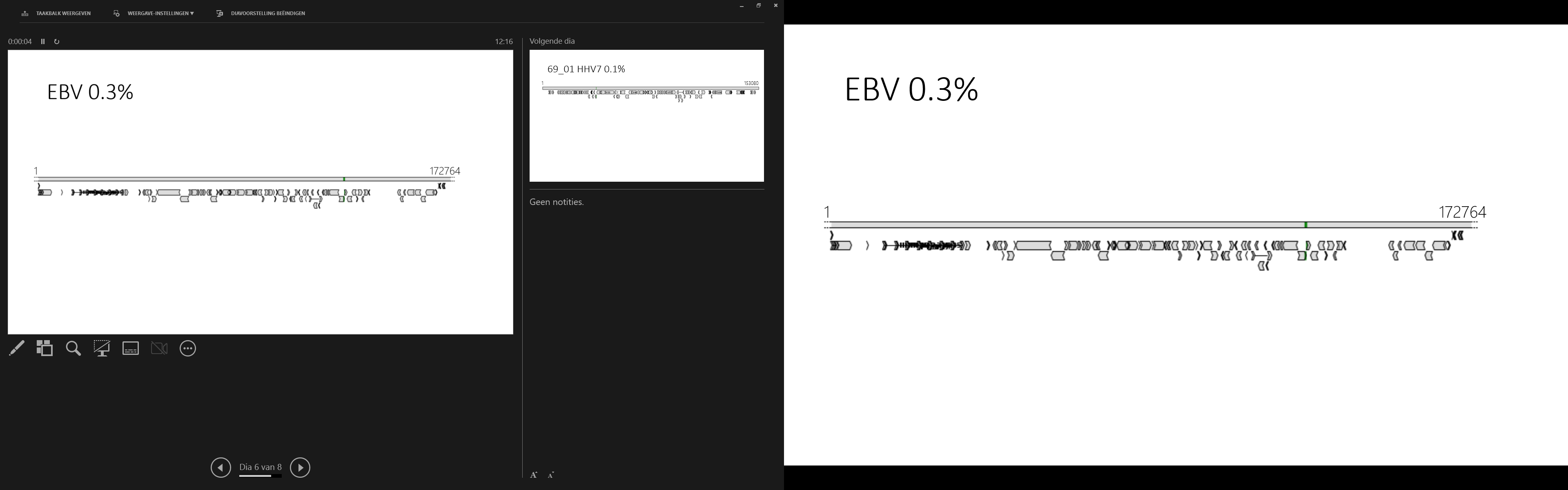 |
| Patient 4 | GenomeDetective:  EBV 237,721 reads, 11.4% coverage, PCR Ct-value 32.3  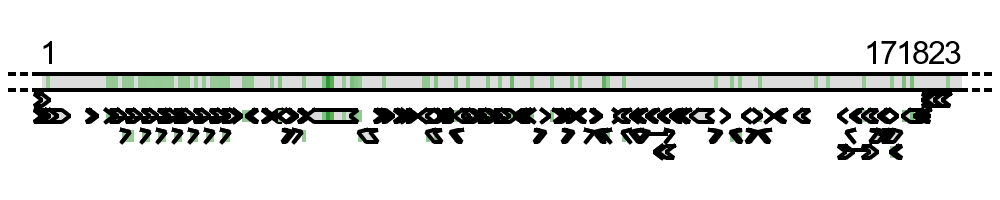 | No biopsy material available |
| Patient 5 | GenomeDetective:  AAV-2 14,937,373 reads, 98.8% coverage, PCR Ct-value 26  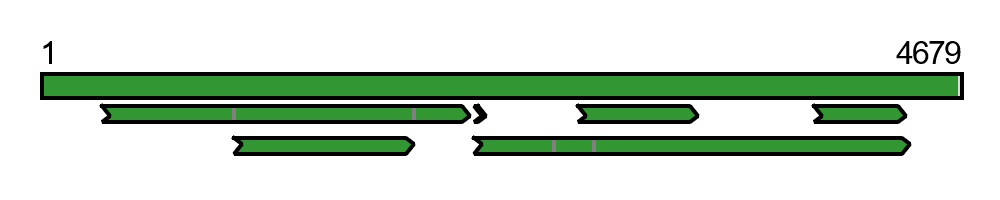  VZV 3,244,606 reads, 16.6% coverage, PCR Ct-value 30.1  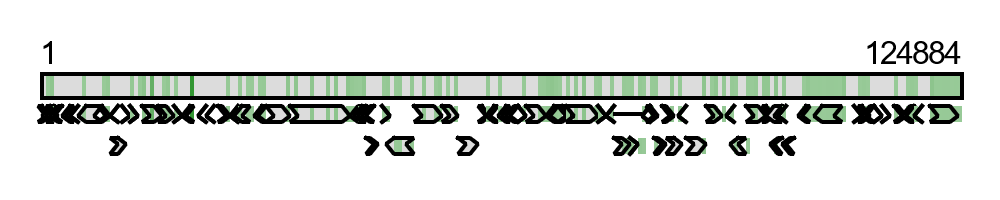  HPyV 747 reads, 1,8% coverage  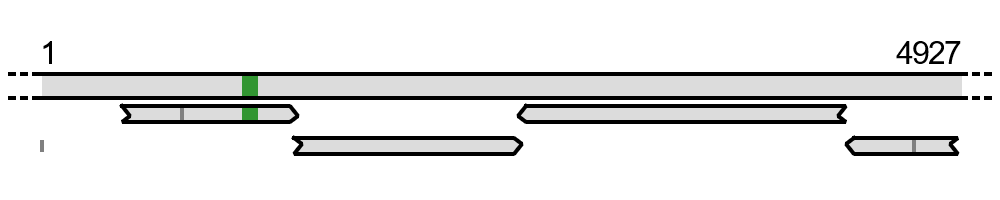  Centrifuge^1^:  TTV10 8,512 reads  PCR Ct-value 28.6, 6.5 log_10_ c/ml  Bowtie2/AgaAligner 1.4% coverage^2^  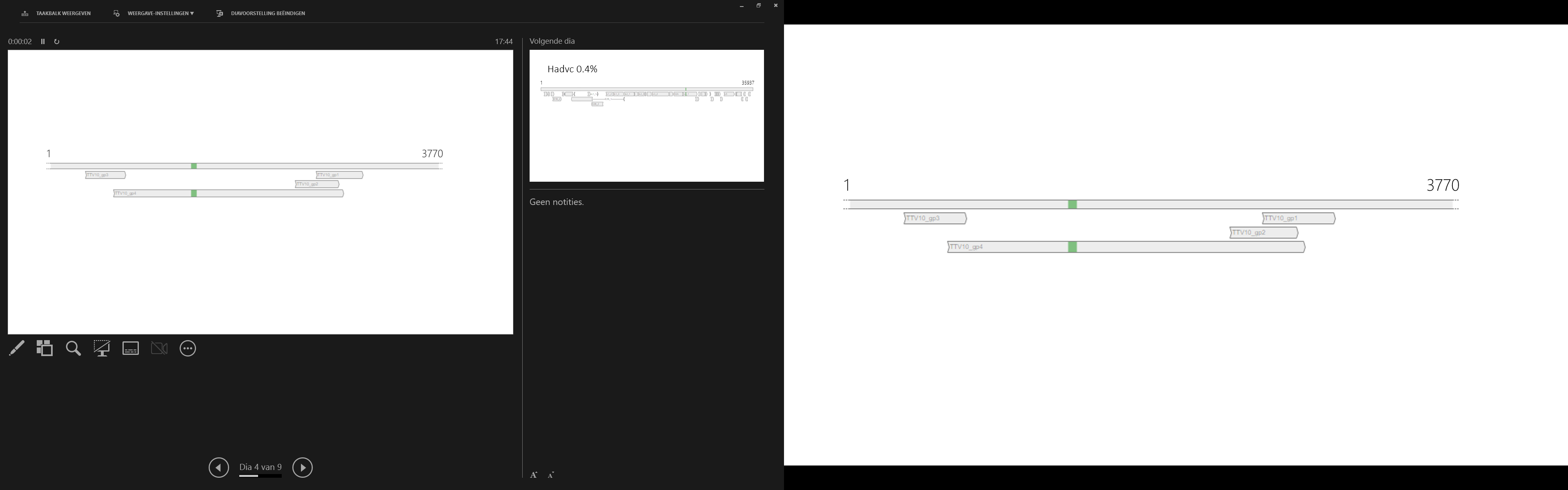 | GenomeDetective:  AAV-2 464,469 reads, 99.4% coverage  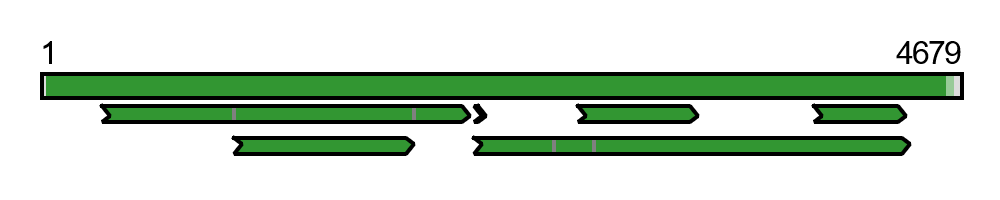  HHV6 7,593 reads, 16% coverage, PCR Ct-value 28.7  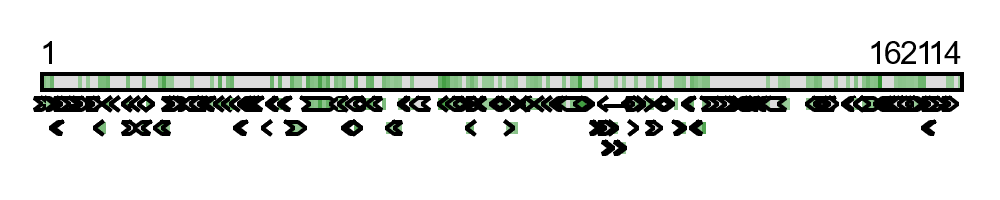  TTV5 301 reads, 25.5% coverage, PCR Ct-value 30.5, 5.9 log_10_ c/ml  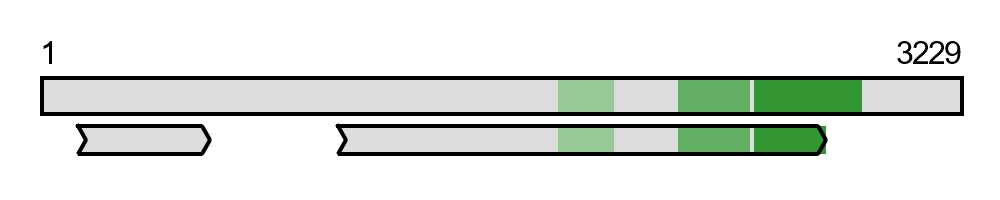  HAdV40/41 104 reads, 0.8% coverage, PCR Ct-value 34.5  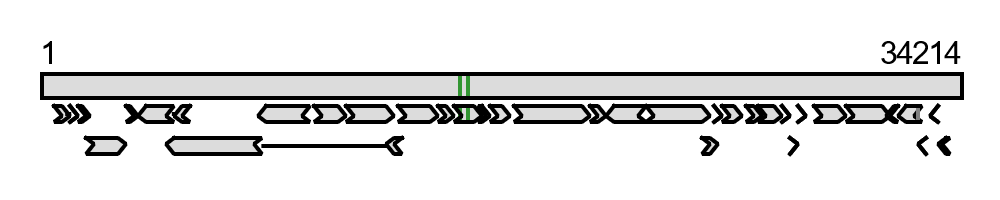 |

^1^ No coverage bars available by Centrifuge classification itself, coverage calculated and visualised by Bowtie2/AgaAligner

^2^ No residual material available for PCR testing,

^3^ Low coverage in combination with PCR Ct-value <30 is likely explained by genomic heterogeneity with short stretches of nucleotide being conserved (doi: [10.1128/JVI.02472-10](https://eur03.safelinks.protection.outlook.com/?url=https%3A%2F%2Fdoi.org%2F10.1128%2FJVI.02472-10&data=05%7C02%7CJ.J.C.de_Vries%40lumc.nl%7C06796908f19e4a87745e08dd15554689%7Cc4048c4fdd544cbd80495457aacd2fb8%7C0%7C0%7C638690178222881410%7CUnknown%7CTWFpbGZsb3d8eyJFbXB0eU1hcGkiOnRydWUsIlYiOiIwLjAuMDAwMCIsIlAiOiJXaW4zMiIsIkFOIjoiTWFpbCIsIldUIjoyfQ%3D%3D%7C0%7C%7C%7C&sdata=XvrzeWRTjbaY630HehhS1gi9MVKHdQfi77hCc%2F3aPpU%3D&reserved=0),DOI: [10.1007/s705-002-8301-7](https://eur03.safelinks.protection.outlook.com/?url=https%3A%2F%2Fdoi.org%2F10.1007%2Fs705-002-8301-7&data=05%7C02%7CJ.J.C.de_Vries%40lumc.nl%7C06796908f19e4a87745e08dd15554689%7Cc4048c4fdd544cbd80495457aacd2fb8%7C0%7C0%7C638690178222904272%7CUnknown%7CTWFpbGZsb3d8eyJFbXB0eU1hcGkiOnRydWUsIlYiOiIwLjAuMDAwMCIsIlAiOiJXaW4zMiIsIkFOIjoiTWFpbCIsIldUIjoyfQ%3D%3D%7C0%7C%7C%7C&sdata=cXVyBWY%2B7JilDY2dZQuclMUiSuahxq%2Fz2ObxfCsDTP0%3D&reserved=0))

**b**


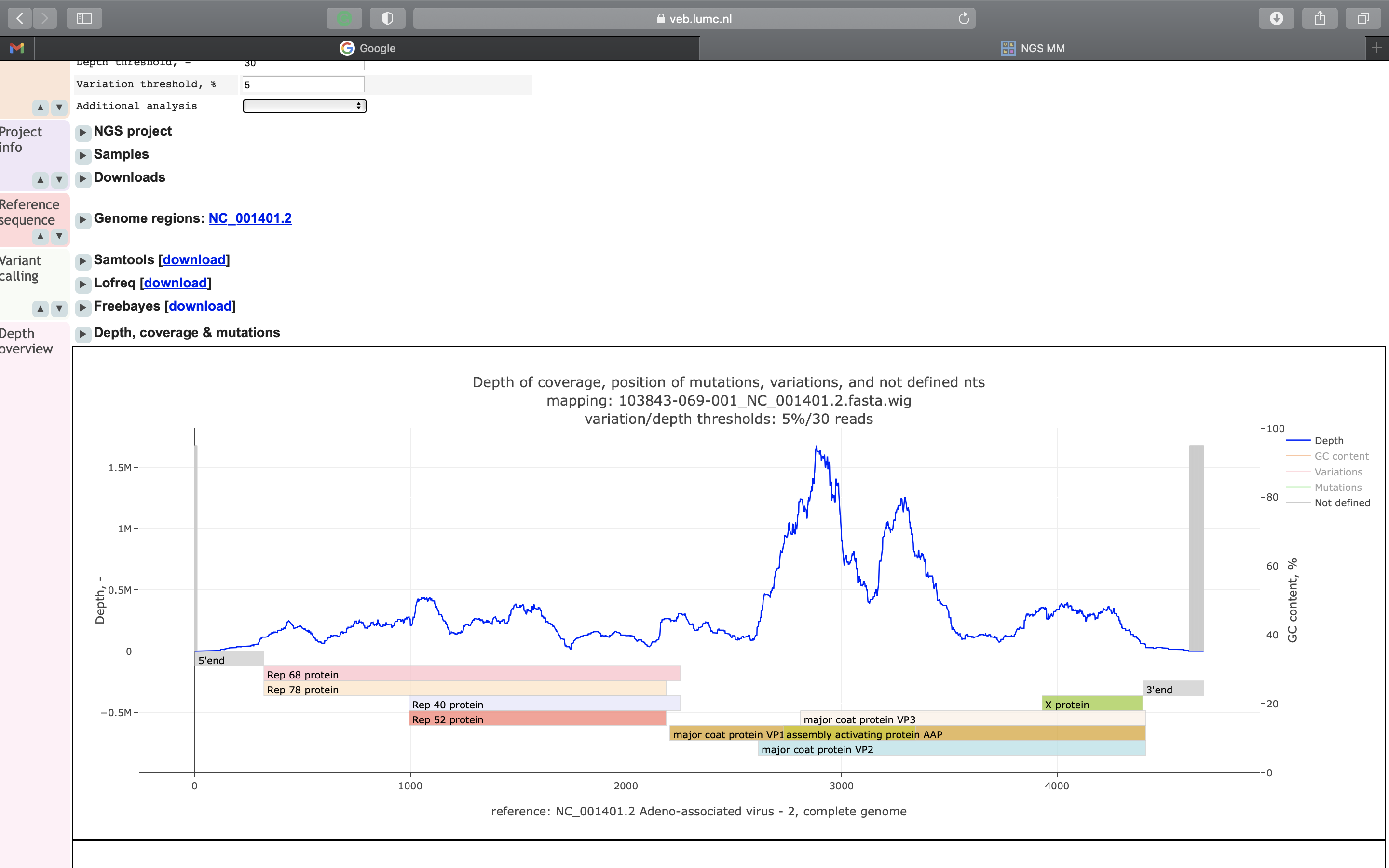

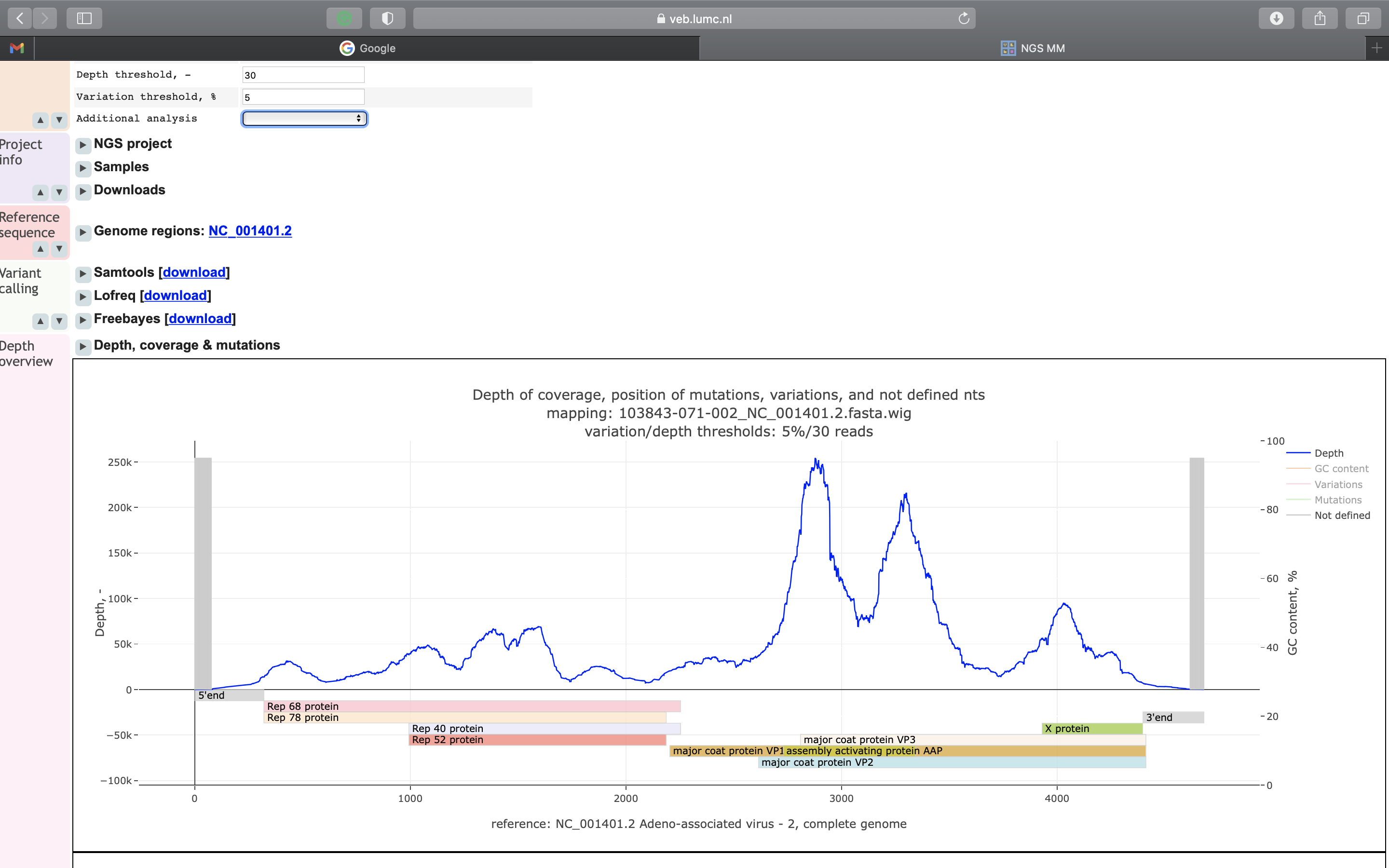


Patient 2 Plasma

Patient 2 Liver

(bp)

(bp)

Coverage: 98.40%, Ct-value 24

Coverage: 97.03%, Ct-value 18


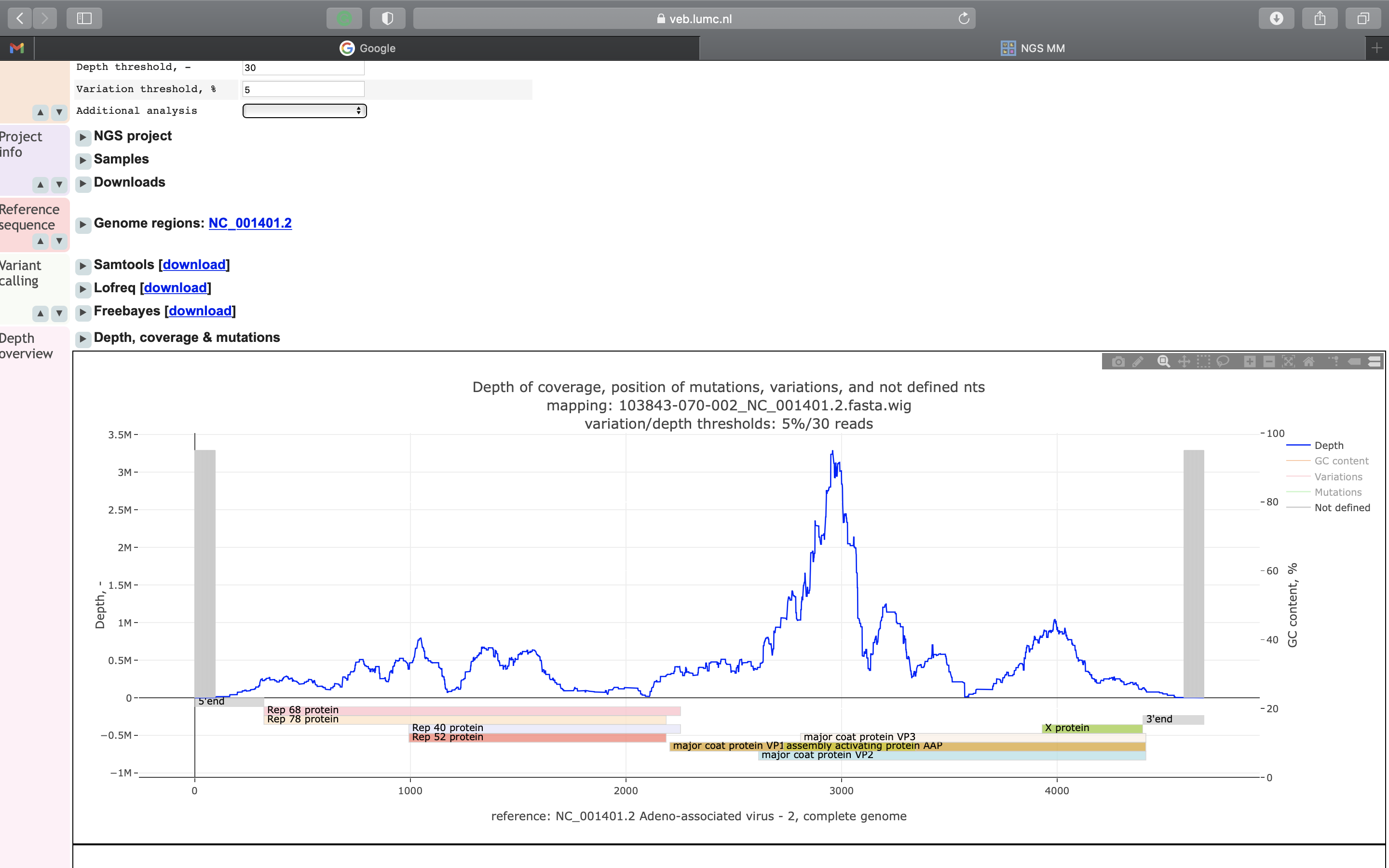

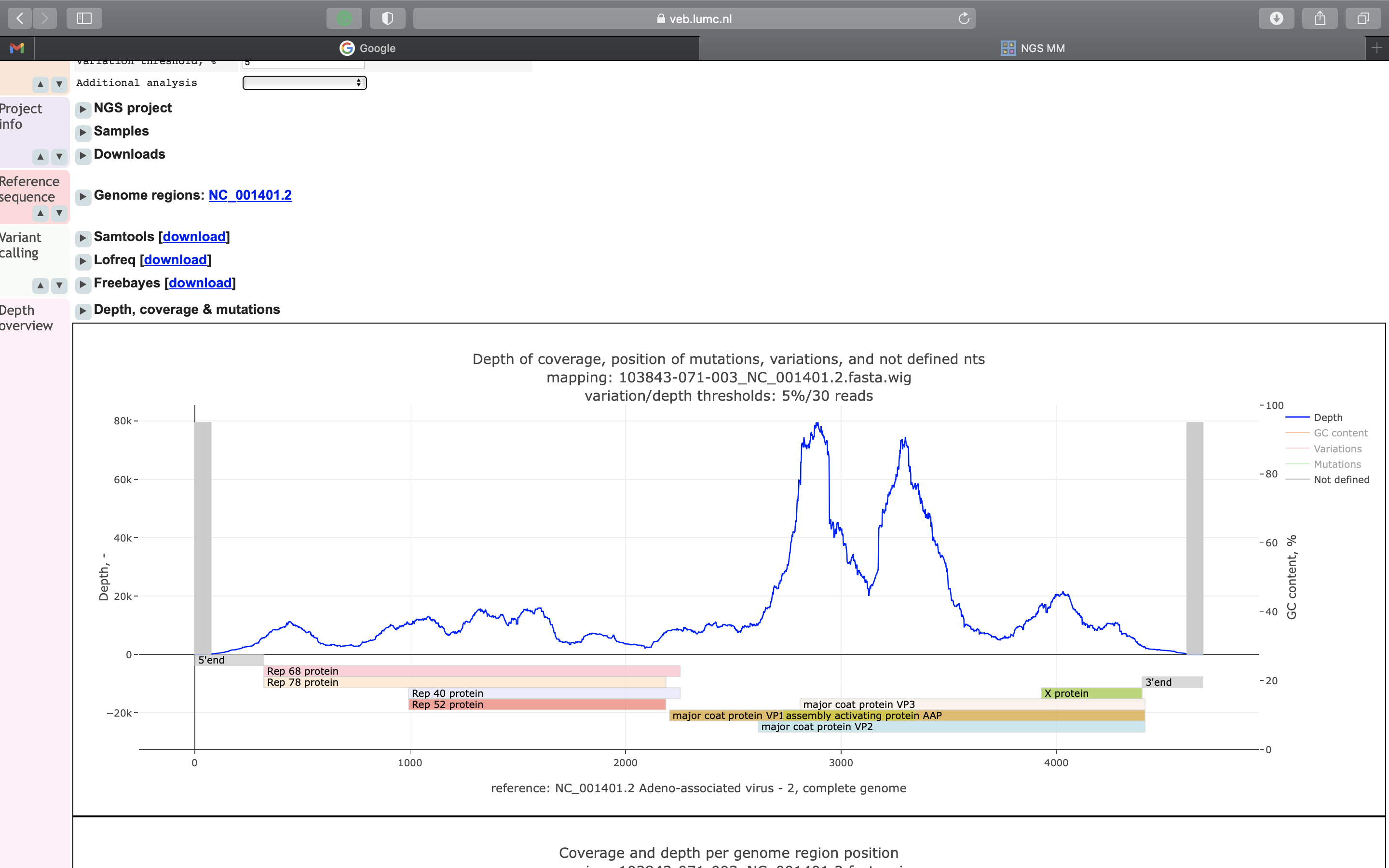


Patient 5 Plasma

Patient 5 Liver

(bp)

(bp)

Coverage: 96.82%, Ct-value 20

Coverage: 96.05% Ct-value 26

**Sequence read coverages plots of main virus findings and of AAV2 genome from paediatric cases of acute liver failure. a,** Results of read counts (unnormalized) and coverage plots of GenomeDetective mapping preceded by binning of viral reads and assembly. **b**, Mapping of all sequence reads obtained from cases by viral metagenomics, using dedicated Bowtie2 mapping tool, to AAV2 reference genome NC_001401.2.
