# Supplementary material for: Metagenomic and transcriptomic investigation of pediatric acute liver failure cases reveals a common pathway predominated by monocytes
Source: mBio. 2025 Mar 18;16(4):e03913-24. doi: 10.1128/mbio.03913-24 (PMC11980388; doi:10.1128/mbio.03913-24)

Supplementary Figure 2. Protein expression as measured in liver tissue lysates of paediatric cases and controls.

a

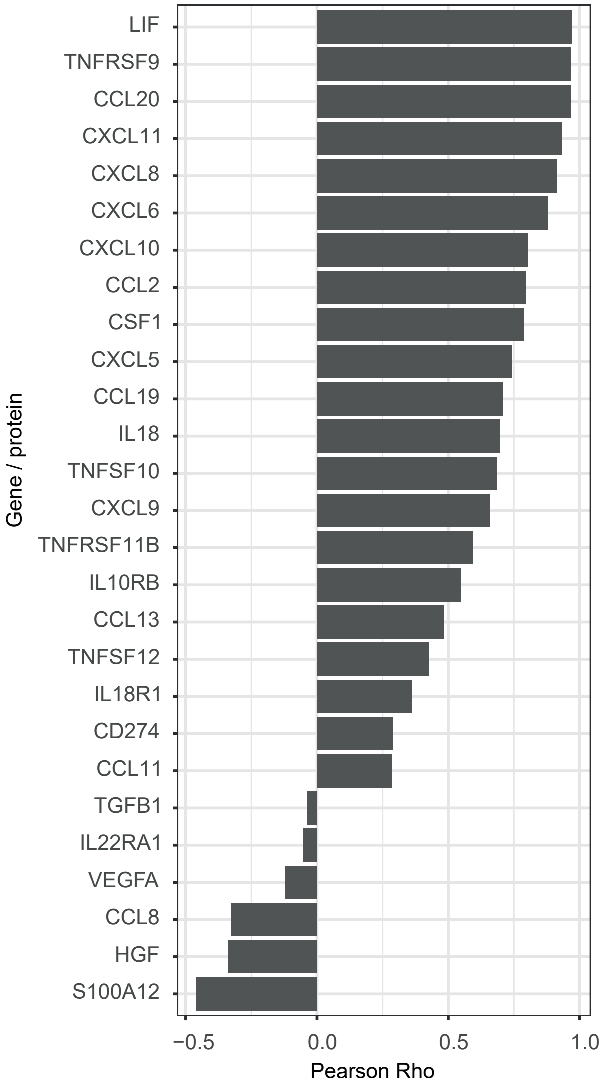

b

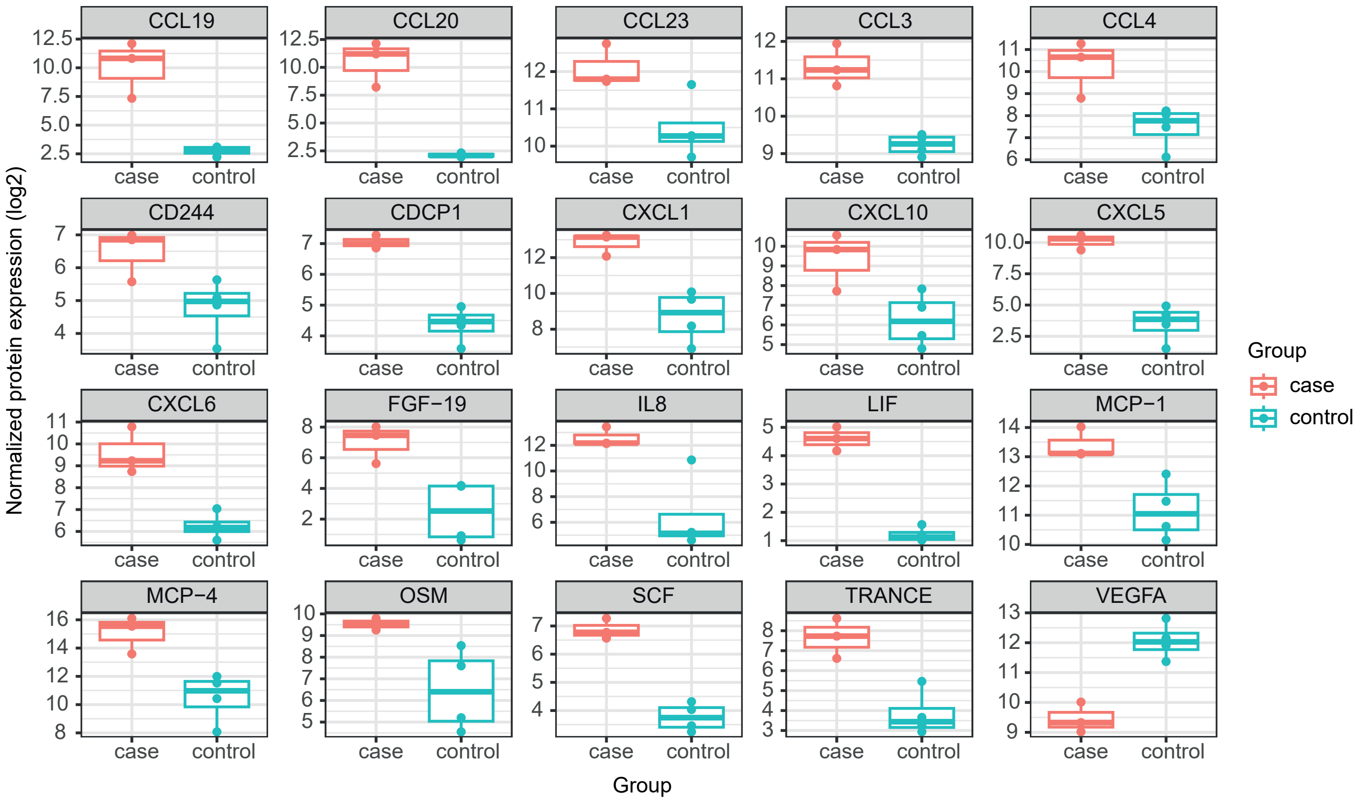

Supplement: Figs S2 — Protein expression as measured in liver tissue lysates of pediatric cases and controls. [file mbio.03913-24-s0002.pdf]
