# Supplementary material for: Metagenomic and transcriptomic investigation of pediatric acute liver failure cases reveals a common pathway predominated by monocytes
Source: mBio. 2025 Mar 18;16(4):e03913-24. doi: 10.1128/mbio.03913-24 (PMC11980388; doi:10.1128/mbio.03913-24)
Supplement: Table S1 — Main clinical characteristics of paediatric acute liver failure cases and pediatric controls. [file mbio.03913-24-s0003.docx]

**Supplementary Table 1. Main clinical characteristics of paediatric acute lever failure cases and paediatric controls.**

**Diagnostic Work-Up and Clinical Characteristics of Pediatric Acute Liver Failure Cases, adapted with permission^2^**

| **Parameter** | **Case 1** | **Case 2** | **Case 3** | **Case 4** | **Case 5** |
| --- | --- | --- | --- | --- | --- |
| **Age** | 11 months | 3 years | 8 years | 17 months | 2 years |
| **Sex** | Male | Female | Female | Female | Female |
| **Past Medical History** | None | None | None | None | Cow's milk allergy |
| **Medication Use Prior** | None | None | None | None | None |
| **Medications Before Referral** | Acetaminophen (stopped upon PALF detection) | Vitamin K | Vitamin K | Ursodeoxycholic acid, Vitamin K | Acetaminophen (stopped upon PALF detection) |
| **Presenting Symptoms** | Vomiting, diarrhea, anorexia, drowsiness | Vomiting, jaundice, acholic stools | Vomiting, jaundice, itching, acholic stools | Vomiting, fever, jaundice, acholic stools | Jaundice, abdominal pain, fatigue, period of vomiting 4 week prior |
| **Days between onset of (GI) symptoms and admission (pALF)/tansplantation** | 4/5 | 28/30 | 14/18 | 2/NA (not transplanted) | 3/16 |
| **Peak/admission AST** | 16312/10310 | 8548/8301 | 1931/1931 | 3880/3880 | 5044/4899 |
| **Peak/admission ALT** | 12320/7439 | 4617/4502 | 1761/1761 | 5597/5597 | 3680/3872 |
| **Peak/admission INR** | 9.0/5.7 | 4.9/2.5 | 4.5/1.7 | 5.8/5.8 | 7.6/1.3 |
| **Etiology - Hepatitis A-E** | Negative for all types | Negative for all types | Negative for all types | Negative for all types | Negative for all types |
| **Adenovirus** | Positive (feces Ct 28, plasma Ct 38) | Positive (plasma Ct 38) | Negative | Negative | Positive (NPA Ct 31, feces Ct 38 after 5 days admission) |
| **SARS-CoV-2** | Positive (NPA Ct 25) | Negative (IgG positive for prior infection) | Negative (IgG positive for prior infection) | Negative (IgG spike negative) | Positive (prior infection IgG positive) |
| **Other viruses detected** | Positive EV (NPA Ct 25, EV feces Ct 31) Negative (plasma) | Negative | Negative | EBV positive (IgM and IgG VCA positive, IgG EBV NA Negative, plasma 3.21 log IU/ml) | VZV Positive (plasma Ct 28, IgG antibodies negative) |
| **Toxicological Screen** | Acetaminophen in therapeutic range | Negative for paracetamol | Not applicable | Acetaminophen in therapeutic range | Not applicable |
| **HLA typing** | No HLA-DRB1*04:01, DQA1*03:03 or DRB4*01:03 | Positive for DRB4*01, DRB1*04, DQA1*03 | No HLA-DRB1*04:01, DQA1*03:03 or DRB4*01:03 | No HLA-DRB1*04:01, DQA1*03:03 or DRB4*01:03 | Positive for DRB4*01, DRB1*04, DQA1*03 |
| **Outcome** | Transplantation (living donor) | Transplantation (living donor) | Transplantation (living donor) | No transplantation needed | Transplantation (living donor) |
| **Survival** | Yes | Yes | Yes | Yes | Yes |
|  |  |  |  |  |  |
|  |  |  |  |  |  |
|  |  |  |  |  |  |
|  |  |  |  |  |  |
|  |  |  |  |  |  |
|  |  |  |  |  |  |
|  |  |  |  |  |  |
|  |  |  |  |  |  |
|  |  |  |  |  |  |
|  |  |  |  |  |  |
|  |  |  |  |  |  |
|  |  |  |  |  |  |
|  |  |  |  |  |  |
|  |  |  |  |  |  |
|  |  |  |  |  |  |
|  |  |  |  |  |  |

Abbreviations: ANA; Anti Nuclear Antibodies, CMV; Cytomegalovirus, Ct; cycle threshold, EBV; Epstein‐Barr virus, EBNA; Epstein Barr virus nuclear antigen, EV; Enterovirus, HHV; human herpes virus, HLA; Humane Leucocyte Antigens, HSV; herpes simplex virus, IgG; immunoglobuline G, NPA; nasopharyngeal aspirate, PALF; pediatric acute liver failure, SARS‐COV; Severe Acute Respiratory Syndrome Coronovirus, VCA; viral capsid antigen, VZV; varicella zoster virus, AST; aspartate aminotransferase, ALT; alanine transaminase. Peak AST, ALT and INR levels were determined up to the start of surgery, if applicable. HLA typing was performed as part of routine transplant screening protocol using Lifecodes HLA RAPID-PCR-SSOP typing kits.

**Characteristics of Pediatric Controls**

| **Parameter** | **Control 6** | **Control 7** | **Control 8** | **Control 9** | **Control 10** | **Control 11** |
| --- | --- | --- | --- | --- | --- | --- |
| **Age** | < 1y | 1-5 y | 1-5 y | 1-5 y | 15-18 y | 15-18 y |
| **Past Medical History** | None | None | None | None | None | None |
| **Type of material available in biobank*** | Postmortem obtained liver biopsy | Postmortem obtained liver biopsy | Postmortem obtained liver biopsy | Postmortem obtained liver biopsy | Postmortem obtained liver biopsy | Postmortem obtained liver biopsy |
| **Event** | SIDS | SUDC | SUDC | SUDC | SUDC | SUDS |
| **Etiology, retrospectively determined** | Acute bacterial sepsis, Hib | Acute bacterial sepsis, GAS | Febrile illness, post VZV | Febrile illness, B19 | Cardio-vascular event | Cardio-vascular event |
| **Time period of biopsy** | 2021 | 2022 | 2018 | 2018 | 2020 | 2020 |

*No plasma/serum samples available in biobank. SIDS; sudden infant death syndrome, SUDC; sudden unexpected death in childhood, B19; human parvovirus B19
